# The Roles of Cancer Stem Cells and Therapy Resistance in Colorectal Carcinoma

**DOI:** 10.3390/cells9061392

**Published:** 2020-06-03

**Authors:** Plabon Kumar Das, Farhadul Islam, Alfred K. Lam

**Affiliations:** 1 Department of Biochemistry and Molecular Biology, University of Rajshahi, Rajshahi 6205, Bangladesh; plabonsb5208@gmail.com; 2 Institute for Glycomics, Griffith University, Gold Coast, QLD 4222, Australia; 3 Cancer Molecular Pathology, School of Medicine, Griffith University, Gold Coast, QLD 4222, Australia

**Keywords:** colorectal carcinoma, cancer stem cells, therapy resistance, signalling pathways, microRNAs, DNA damage

## Abstract

Cancer stem cells (CSCs) are the main culprits involved in therapy resistance and disease recurrence in colorectal carcinoma (CRC). Results using cell culture, animal models and tissues from patients with CRC suggest the indispensable roles of colorectal CSCs in therapeutic failure. Conventional therapies target proliferating and mature cancer cells, while CSCs are mostly quiescent and poorly differentiated, thereby they can easily survive chemotherapeutic insults. The aberrant activation of Wnt/β-catenin, Notch, Hedgehog, Hippo/YAP (Yes-associated protein) and phosphatidylinositol 3-kinase/protein kinase B facilitates CSCs with excessive self-renewal and therapy resistance property in CRC. CSCs survive the chemo-radiotherapies by escaping therapy mediated DNA damage via altering the cell cycle checkpoints, increasing DNA damage repair capacity and by an efficient scavenging of reactive oxygen species. Furthermore, dysregulations of miRNAs e.g., miR-21, miR-93, miR-203, miR-215, miR-497 etc., modulate the therapeutic sensitivity of colorectal CSCs by regulating growth and survival signalling. In addition, a reversible quiescent G_0_ state and the re-entering cell cycle capacity of colorectal CSCs can accelerate tumour regeneration after treatment. Moreover, switching to favourable metabolic signatures during a therapeutic regimen will add more complexity in therapeutic outcomes against CSCs. Therapeutic strategies targeting these underlying mechanisms of CSCs’ therapy resistance could provide a promising outcome, however, deep understanding and concerted research are necessary to design novel therapies targeting CSCs. To conclude, the understanding of these mechanisms of CSC in CRC could lead to the improved management of patients with CRC.

## 1. Introduction

Colorectal carcinoma (CRC) is the second most common cause of cancer-related death worldwide, with an estimated 862,000 deaths recorded in the year 2018 [1]. Patients with colon carcinoma can be managed with surgery alone, whereas patients with rectal carcinoma are often treated by chemotherapy e.g., with 5-fluorouracil (5-FU), followed by surgery [2]. After surgery, the patients with CRC could be managed by chemotherapy or molecular target therapies. In general, 5-FU-based regiment in combination with oxaliplatin are used for stage III CRC (with lymph node metastases), or with stage II CRC with high risk of recurrence after surgery. The first line treatment of CRC with distant metastases includes mFOLFOX6 (folinic acid, 5-fluorouracil, and oxaliplatin), with or without molecular targeted therapies (bevacizumab or cetuximab) depends on the patients’ biological status and presence of molecular markers [2]. The threatening fact is that most patients with advanced CRC eventually surrendered to the disease, despite some initial response to these therapies [2]. Therefore, determining the molecular mechanisms regarding drug resistance is imperative to design novel strategies to treat patients with CRC.

As therapy resistance continues to be the biggest problem in cancer treatment, expectedly, a plethora of underlying mechanisms has been proposed over the last few decades [3]. For example, mutations in key signalling pathway molecules, the increased expression of anti-apoptotic proteins, the presence of quiescent and/or resistant tumour cells or the over-activation of drug efflux pumps are all potential causes of therapy failure [4,5]. On top of that, in the last two decades, attention has been focused on the role of a specific population of cancer cells, namely cancer stem cells (CSCs). The theory of cancer stem cells (CSCs) describes that cancer cells are hierarchically organized, adding a new level of complexity to therapy failure [3]. CSCs have been recognized as the root of cancers initiation and resistance of cancer cells to conventional chemo- and radiotherapies, which could cause cancer recurrence years after therapeutic termination [3,6,7,8]. The differentiated cancer cells or non-CSCs undergo apoptosis upon chemotherapies, whereas the CSCs exhibit resistance to therapeutic insults, thereby escaping the apoptotic cell death [9]. Then, the surviving fraction of CSCs can re-establish their number, which subsequently results in clinical cancer recurrence [9]. CSCs have been found to bypass the therapeutic insults in different cancers, including CRC [9,10,11,12]. In addition, in CRC, the numbers of CSCs increased in xenotransplanted mice after chemotherapy [10]. These CSCs confer resistance to radiotherapy by the selective activation of DNA damage checkpoints [7,9,12]. A growing body of evidence suggests that CSCs in CRC exhibit intrinsic characteristics of therapy resistance and are associated with cancer regeneration and recurrence after conventional therapies [9,10,13,14].

**Figure 1:**
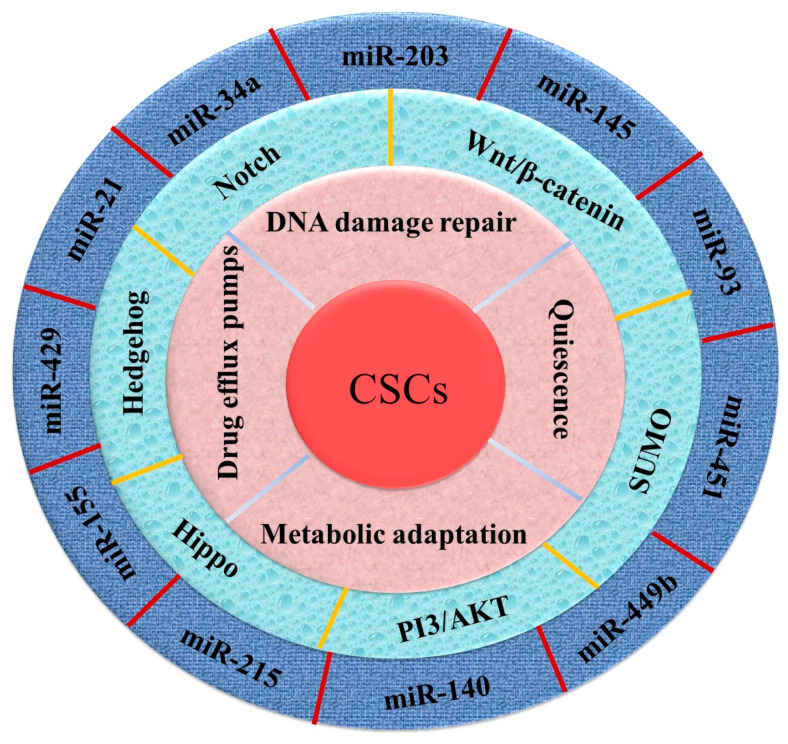
The features represent special characteristics, crucial signalling pathways and associated miRNAs of cancer stem cells (CSCs) in causing therapy resistance in colorectal carcinoma.

The underlying mechanisms of CSCs in CRC attributed to therapy resistance include, the activation of growth signalling pathways such as Hedgehog, Notch, TGF- β, Wnt/β-catenin, Hippo, PI3/AKT etc., the dysregulation of microRNAs, acquisition of quiescent state, metabolic switch, phenotypic plasticity etc. (Figure 1). For instance, the dysregulation of many microRNAs (miRNAs) contributes to the acquisition of chemo-resistant phenotype in colorectal CSCs [15,16,17,18,19,20]. Furthermore, the activation of quiescent state and metabolic switch into more stable phenotype as well as evading drug induced DNA damage collectively power CSCs in CRC to become impregnable [21,22,23]. Considering all of these, in this review, we exemplify the therapy resistance property of colorectal CSCs, and discuss the mechanisms of how this malicious property is activated in colorectal CSCs. Furthermore, we outline some therapeutic options to overcome the therapy resistance of CSCs.

## 2. CSCs: The Ultimate Survivors and Their Therapy Resistance in CRC

The concept of CSCs being the main player in cancer responsible for resistance to conventional therapy originates from multiple observations using cell culture, animal models and patients with cancer. In the cell culture of colon carcinoma, the direct analysis of apoptosis demonstrated that differentiated cancer cells or non-CSCs were induced to death upon chemotherapy. On the other hand, CSCs survived the therapeutic insults [9]. This variation in sensitivity towards therapy did not result from differences in proliferation rates between CSCs and more differentiated cancer cells, because therapy induced deaths are not dependent on the proliferative status of cells [9]. Moreover, these surviving CSCs can effectively re-populate the culture, proving their indispensable role in causing chemotherapy failure and cancer relapse in patients with cancer [9].

Followed by in vitro evidence, escaping from chemotherapy of CSCs was noted in xenograft model in colon carcinoma [24]. An increase of CD133+ tumour cells (CSCs’ phenotype) has been found after oxaliplatin treatment in xenotransplanted colon cancer cells [24]. CD133+ CSCs are more resistant to oxaliplatin in vivo when compared to that of differentiated non-CSCs (CD133- cells). In addition, xenotransplanted mice with cancer cells bearing stem cell surface markers (ESA+ /CD44+ /CD166+) derived from patients with colon carcinoma showed an increased resistance to irinotecan [10]. Furthermore, these CSCs are also resistant to radiotherapy [12]. A CSCs subpopulation derived from human colon adenocarcinoma (HT-29) cells had shown an increased resistance to radiotherapy [25]. Thus, the elimination of CSCs could significantly reduce cancer recurrence, metastasis and chemo-resistant phenotype [3].

## 3. Dysregulation of Growth Signalling Pathways: Epicentre of Therapy Resistance in CSCs?

**Figure 2:**
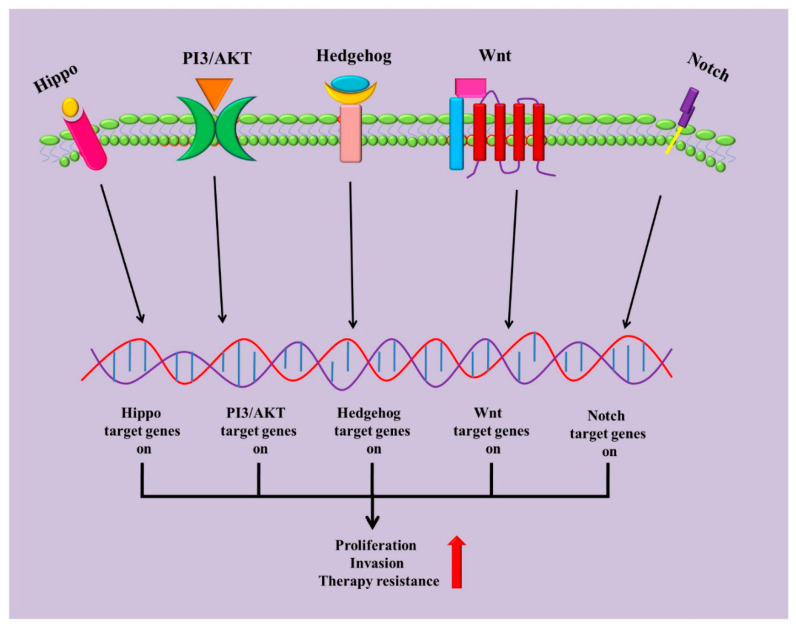
Overview of signalling pathways defining colorectal CSC populations. Wnt, Notch, Hedgehog, PI3/AKT and Hippo pathways are implicated to regulate CSC populations, leading to increase in proliferation, invasiveness, and most importantly, resistance to therapies.

Current conventional adjuvant therapies target predominately more differentiated cancer cells within a cancer. These treatments fail to recognize and kill the CSCs, which result in therapy escape/resistance and thereby, cancer recurrence [19]. Aberrant activations of growth and survival signalling pathways in CSCs [15,16,17,18,19,26] have been attributed to therapy resistance of CRC (Figure 2). In the intestinal crypt, Wnt signalling plays a crucial role in the self-renewal of CSC in CRC [27]. Activation of Wnt signalling facilitates both the differentiation and proliferation of CSCs [28]. In addition, the activation of Wnt/β-catenin signalling targets *Lgr5*, which is a primary marker of CSCs in the intestinal crypt [29]. Other putative CSC markers, including CD44, CD24, CD133, ATP-binding cassette (ABC)transporters, and EpCAM are direct targets in the Wnt signalling pathway [14,30,31,32,33]. All these markers are strongly associated with the therapy resistance property of CSCs.

More than 90% of classical mutations in Wnt/β-catenin signalling (i.e., APC truncations, β-catenin mutations) in CRC are different from other cancer types [34]. Thus, therapeutics targeting upstream factors of Wnt/β-catenin signalling pathways may fail to suppress the aberrant gene transcription caused by the mutations [34]. Most of the mutations in the Wnt/β-catenin signalling pathway in CRC eventually cause the accumulation of β-catenin, which subsequently results in the transcription of target genes, thereby increased cellular growth and survival [34]. Furthermore, exosomes (extracellular vesicles that contain constituents like protein, DNA, and RNA) derived from fibroblasts promote chemoresistance in patients with CRC, by activating stem cell like traits with the help of Wnt signalling [35]. In addition, fibroblasts confer chemo resistance in CRC via exosome-induced reprogramming (dedifferentiation) of differentiated cancer cells to phenotypic and functional CSCs. Wnts signalling mediates the major reprogramming regulators in exosomes derived from fibroblasts. Exosomal Wnts increased Wnt activity and drug resistance in differentiated CRC cells, and expectedly, inhibiting Wnt release demolished this effect in both in vitro and in vivo [35]. Therefore, exosomal Wnts derived from fibroblasts could induce the dedifferentiation of cancer cells to promote chemo resistance, suggesting that the inhibition of exosomal Wnt signalling might be useful to improve chemo sensitivity of CRC via suppressing the CSCs like phenotype.

Notch signalling is another pathway that has been implicated in the growth of CSCs and contributes in therapy resistance in patients with CRC [17]. Higher Notch1 expression was noted in the colonospheres and chemo-resistant cells derived from colon carcinoma cells (HCT116), when compared with that of parental cells. The colonospheres and chemoresistant cells proliferated at a significantly slower rate than parental cells [17]. Parental cells were sensitive to 5-FU and Oxaliplatin when compared with the untreated cells, however, colonospheres and chemoresistant cells were resistant to 5FU and Oxaliplatin when compared to the parental cells. The Notch signalling pathway, particularly Notch1, was reported to contribute significantly in the generation of colonospheres and chemoresistant cells, and the activation of Notch was involved in maintaining the CSCs-like phenotypic characteristic. Importantly, the inhibition of Notch signalling in vitro by DAPT (Notch pathway inhibitor) was found to cause a decrease in cell growth and reduced the number of colonospheres and chemoresistant cells [17]. Therefore, Notch signalling may be important for the maintenance of CSC and resistance to standard chemotherapeutics in cancer.

Hedgehog signalling regulates the anti-cancer drug resistance of human colon cancer from patient-derived air liquid interface (ALI) organoids [36]. GLI-1 (nuclear mediator of Hedgehog signalling) is a component of Hedgehog signalling which plays a vital role in carcinogenesis and the resistance to therapy resistance in CRC [36,37,38]. For example, GLI-1 expression was enhanced in 5-FU resistant cancer cells derived from colon cancer cells (LoVo) when compared with non-resistant cells. In addition, the knockdown of *GLI-1* gene decreased the resistance of cells to 5-FU [39]. Furthermore, in air liquid interface (ALI) organoids derived from patients with colon cancer, Hedgehog signal inhibitor reduced the resistance to 5-FU, Irinotecan and Oxaliplatin via the inhibition of GLI-1 expression [39]. Treatment with Hedgehog signal inhibitors (AY9944, GANT61) decreased the cell viability of organoids. Chemotherapeutic drugs, such as 5-FU, Irinotecan or Oxaliplatin, could reduce the cell viability of tumour organoids when combined with Hedgehog inhibitors (AY9944 or GANT61). Moreover, treatment with AY9944 or GANT61resulted in the inhibition of expression of other stem cell markers such as c-Myc, CD44 and Nanog, through reduction of the expression of transcription factor GLI-1 [39].

Hippo/YAP (Yes-associated protein) signalling is a potential pathway, which regulates tissue homeostasis, organ size and stem cells [40]. YAP1 (Yes-associated protein 1) signalling is associated with cell proliferation and metastasis in CRC [40]. Higher expression of YAP target genes in the tumour was coupled with an increased risk of cancer relapse and poor survival in many patients with CRC treated with 5-FU. In addition, the elevated expression of YAP target genes could be a major alteration in the 5-FU resistant colon cancer cells [41]. Accordingly, knockdown of YAP1 sensitized 5-FU resistant cells to 5-FU treatment, both in vivo and in vitro. Tyrosine kinase YES1 is known to regulate drug resistance through the regulation of YAP1, which was up-regulated in the 5-FU resistant cells [41]. Several possible causes of YAP1 signalling mediated 5-FU resistant in CRC have been proposed, which induce stemness and quiescence in CRC (as CSC phenotype). Underlying mechanisms of these changes include the increased activation of receptor tyrosine kinases (RTKs), epithelial-mesenchymal transition (EMT) and the elevated expression of YAP1 itself. Furthermore, results from large number of patients with CRC suggested that high expression of YAP1, TEA domain family member 2(TEAD2) and YAP1 target genes *cysteine-rich angiogenic inducer 61* (*CYR61*) and *ankyrin repeat domain 1* (*ANKRD1*) were associated with a high risk of cancer relapse [2].

Phosphatidylinositol 3-kinase/protein kinase B (PI3K/AKT) is a prominent pathway which serves a crucial role in chemoresistance to cancer [42,43]. The activation of the PI3K/AKT pathway is associated with cancer therapy-associated resistances in many human malignancies, including CRC [44]. Metastasis-associated colon cancer 1 (MACC1) correlates with PI3K/AKT in the development of the resistance of 5-FU and CSC-like properties in cancer cells. *MACC1* was upregulated in 5-FU resistant colon cancer cells. In addition, *MACC1* knockdown enhanced 5-FU sensitivity and reduced multi drug resistant protein 1 (MDR1) protein expression [45]. The knockdown of *MACC1* resulted in decreased sphere formation, and reduced the expression levels of pluripotent markers, CD44, CD133 and Nanog. Most importantly, the activation of the PI3K/AKT signalling pathway is involved in the regulatory effects of MACC1 in 5-FU resistant cancer cells. Lower activated phosphorylated AKT (p-AKT) protein level was noted in the *MACC1*-depleted CRC cells. Furthermore, treatment with LY294002(phosphoinositide 3-kinases inhibitor) to 5-FU resistant colon cancer cells (HCT116) resulted in decreased cell survival rate and sphere formation when compared with untreated 5-FU resistant colon cancer cells [45]. Hence, PI3K/AKT signalling pathway may serve an indispensable role in 5-FU resistance and CSC-like properties via MACC1.

A summary of signalling pathways involved in CSCs induced therapy resistance of CRC is given in Table 1. It is evident that therapeutics targeting different CSC properties via inhibiting the components of growth and survival signalling could have the potential for treatment of patients with CRC.

## 4. Evasion of Drugs Mediated DNA Damage

CSCs in cancer can defend themselves from conventional chemotherapeutic treatment by escaping DNA damage induced by therapies. Consequently, the drugs, which target cellular DNA to induce cell death, include cisplatin, oxaliplatin (DNA cross-linkers), methotrexate (inhibitor of DNA synthesis), doxorubicin and daunorubicin (topoisomerase inhibitors) become insensitive to CSCs [23]. To resist DNA damage, CSCs use multiple mechanisms, including altering the cell cycle checkpoints, increasing DNA damage repair capacity, and protecting DNA damage by an efficient scavenging of reactive oxygen species (ROS), generated by the chemotherapy or radiotherapy [23].

Abnormal DNA damage response and alterations in checkpoint pathways may occur by the actions of chemical and physical agents, e.g., chemotherapeutic drugs, which in turn, can interact with nucleic acids [46]. Alterations in cell cycle checkpoints help cancer cells to react differentially in order to ignore DNA damaging agents. Checkpoint kinases, such as checkpoint kinase 1(Chk1), checkpoint kinase 2 (Chk2), along with p53, regulate cell cycle by phosphorylation and dephosphorylation processes. However, uncontrolled activation and inhibition of these important players may induce therapy resistance property in CSCs of CRC [46]. Moreover, colon CSCs increase the expression of DNA repair gene to enhance DNA repair capacity. For example, *O(6)-methylguanine-DNA methyltransferase*, a DNA repair gene, was found to be overexpressed in CSCs when compared to that of non-CSCs [47]. In addition, an increased proliferation was noted in CSC population when compared to that of non-CSCs cells [47]. Clonospheric populations of CRC exhibited less cell death when compared to parental cells after inducing DNA damage through irradiation, suggesting the possible radio-resistant property of clonospheres [48]. X-rays, a widely used option for radiotherapy, leads to the formation of double-strand breaks (DSBs), which infers DNA damage response. If the double-strand breaks are not efficiently repaired, cells undergo apoptosis [49]. The presence of γH2AX foci per nuclei is indicative of double-strand breaks sites in the DNA. The number of γH2AX foci formed per nuclei was decreased significantly in CSCs-like cells derived from colon cancer cells (HCT116), when compared to parental colon cancer cells after exposure to irradiation [50]. The lower number of γH2AX foci per nuclei in clonospheres upon higher dosage of radiation indicated the presence of an efficient DNA damage repair mechanism in CSCs, thereby contributing to the radio-resistance of the clonospheric population [50]. Similarly, reduced olive tail moment, which represents the product of the percentage of total DNA, was observed in clonospheres when compared to parental cells after irradiation [50]. ATM is a protein kinase with a driving role in the DNA damage response, phosphorylates γH2AX and promotes double-strand breaks repair [50]. Active phosphorylated ATM (p-ATM) expression in clonospheres after irradiation was significantly higher, which contributes to DNA damage repair [48,51]. Efficient DNA damage repair mechanisms in clonospheres were further analysed by studying the expression of excision repair cross-complementation group 1(ERCC1). ERCC1is a protein involved in the nucleotide excision repair (NER) pathway, and is a prognostic factor in patients with CRC [48]. ERCC1 mRNA and protein expressions were enhanced with increasing dosages of radiation in clonospheres when compared to parental cells [48]. Therefore, the low level of γH2AX foci per nuclei coupled with ERCC1 expression upon radiation exposure suggest the ERCC1 mediated activation of DNA damage repair machinery in colorectal CSCs.

G9a (histone methyltransferase) is involved in DNA damage response, leading to the malignant phenotype of CRC [52,53]. It is a protein lysine methyltransferase involved in histone methylation, which in turn regulates gene expression, thereby promoting CSCs via chemoresistance and epithelial-mesenchymal transition (EMT) [54,55]. The depletion of G9a suppresses tumour initiation and induces the expression of protein phosphatase2A (PP2A). PP2A expression resulted in attenuation of DNA damage response, and thereby sensitized cancer cells to chemo-radiotherapy [56]. G9a is a potential therapeutic option in eradicating CSCs in colon carcinoma, especially in patients with stage II cancers. It could be used as a predictive biomarker. A significantly higher level of G9a expression was observed after radiation. A positive correlation between G9a and CD133 (CSC marker) was noted in patients with locally advanced rectal cancer having neoadjuvant chemoradiotherapy. Accordingly, the knockdown of G9a resulted in cell population with high sensitivity to radiation treatment and to other DNA damaging agents through PP2A-RPA (replication protein A) axis. Therefore, G9a could act as a novel target in the prediction of the response to chemo-radiotherapy in patients with advanced CRC and response to neoadjuvant chemoradiation therapy in patients with rectal carcinoma [56].

Higher DNA damage repair capacity and higher free radical scavenging potentials could simultaneously make colorectal CSCs more resistant to cancer therapies [13]. For instance, CSCs had shown resistance to genotoxic stress by lowering the production of reactive oxygen species (ROS) and the scavenging of already produced ROS after therapy [57]. The increased activation of genes involved in ROS scavenging, including superoxide dismutase, glutathione peroxidase and catalase in CSCs, indicated that CSCs revive DNA-damaging therapies by reducing toxic insults imposed by treatments through more efficient ROS scavenging [57]. Subsets of CSCs in some human cancers possess lower ROS levels compared to non-tumorigenic cells. Since ROS is a critical mediator of radiation-induced cell killing, CSCs in these cancers develop less DNA damage and are preferably spared after irradiation compared to non-tumorigenic cells [57]. The pharmacological intervention of ROS scavenging property in CSCs could significantly decrease their clonogenicity and resulted in increased in sensitization of cancer cells to radiotherapy [57]. Overall, CSCs with lower ROS levels and enhanced ROS defence, when compared to their non-tumorigenic progeny, contribute to tumour radio-resistance.

## 5. MicroRNAs as Regulators of CSC Therapy Resistance in CRC

**Figure 3:**
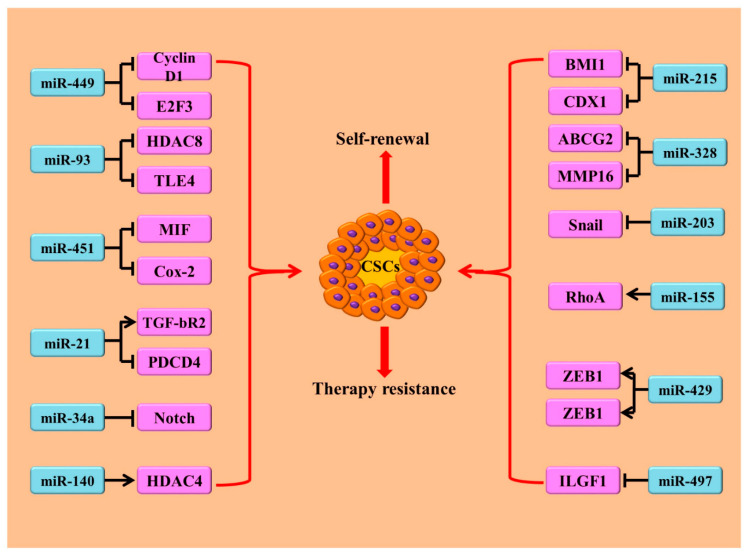
miRNAs in regulating characteristics of CSCs in colorectal carcinoma (CRC): miRNAs can both inhibit or stimulate the development and characteristics of CSCs, such as the ability of self-renewal, therapy resistance, etc. These changes are through targeting signalling pathways, transcription factors, proteins associated with cell cycle, drug efflux, apoptosis and histone deacetylation, etc.

MicroRNAs (miRNAs) play a key role in the regulation of cancer cells with intrinsic/acquired drug resistance through various mechanisms [17,58,59,60,61]. Several miRNAs modulate the sensitivity of CSCs to anti-cancer therapies. Thus, they have been involved in CSCs mediated therapy resistance in CRC (Table 2). As miRNAs regulate cellular growth, proliferation and survival by modulating target genes’ expression, they may contribute to resistance to chemotherapies by modulating specific signalling pathways in colorectal CSCs [Figure 3]. Although the roles of miRNAs in colon CSCs are not fully understood, studies revealed that several miRNAs have been involved in the regulation of CSCs in CRC [17,59,60,61,62,63,64,65,66]. miRNAs regulate the characteristics of CSCs by directly or indirectly targeting the signalling pathways or their components implicated in CSCs. There were differential expressions of 62 miRNAs noted on the analysis of miRNA profiles of CSCs (CD133+/CD44+) and non-stem cells (CD13−/CD44−) from colon adenocarcinoma (SW116) [59]. Among these 62 miRNAs, 31 were up-regulated in colon CSCs and the other 31 miRNAs were down-regulated [59]. One of these miRNAs, miR-449b, is a tumour suppressor and inhibits CSCs proliferation by targeting cyclin D1 (CCND1) and E2F3 transcription factor [60]. Similarly, miRNA expression profiles of colon CSCs (CSC in SW1116) and parental cancer cells revealed that35 miRNAs were up-regulated, whereas 11 miRNAs were down-regulated in CSCs. The restoration of down regulated miR-93 expression in CSC population resulted in inhibition of cell proliferation and colony formation by targeting histone deacetylase 8(HDAC8) and the transducing-like enhancer of Split (TLE4), thereby inhibiting the Wnt/β-catenin signalling pathway [61].

Other studies also noted the differential expression of miRNAs in colon CSCs (CD133+ population) and non-CSC (CD133- population) colon carcinoma cells [63,64,65,66]. These differential expressed miRNAs, such as miR-451, miR-429 and miR-155, are associated with the differentiation and EMT process in CSCs [65,66]. Interestingly, miR-451 was down regulated in irinotecan resistant colonspheres and an exogenous over expression of miR-451 sensitized colonspheres to irinotecan, along with the reduction of self-renewal and tumourigenicity [66]. miR-451mediates its anti-tumourigenic activity by directly targeting the expression of macrophage migration the inhibitory factor (MIF) gene, thereby indirectly modulating the cyclooxygenase-2 (COX-2) expression, which in turn regulates the activity of Wnt signalling and controls the growth of colon CSCs [66].

miR-21, one the most abundant and vigorously studied oncogenic miRNAs in many tumours, is involved in CSCs-enrichment and chemoresistant in colon carcinoma cells [67,68]. For instance, the down-regulation of miR-21 could enhance the susceptibility of therapeutic regimens, whereas the up-regulation of miR-21 causes the activation of Wnt signalling pathway and its targeted genes *c-Myc* and *cyclin D1* (*CCND1*). Furthermore, miR-21 mediates its action through targeting transforming growth factor, beta-receptor 2 (TGF-βR2), thereby down-regulating programmed cell death (PDCD4) [67]. In addition, the expression of miR-21 is associated with the expression of another miRNA miR-145, a miRNA associated with CSCs-enrichment in CRC cells. Interestingly, miR-21 and miR-145 have reciprocal action in cancer. Replacement of mature miR-145 or treatment with antagomiR-21 (antisense miR-21) resulted in the suppression of the growth of colon carcinoma in severe combined immunodeficiency (SCID) xenograft mice [68]. Therefore, the cooperation of miR-21 and miR-145 in regulating CSCs proliferation and/or differentiation might dictate the development of chemoresistance in colorectal carcinoma. Similarly, miR-34a, another widely recognized tumour suppressor miRNA, regulates the proliferation and differentiation of colorectal CSCs by targeting Notch signalling [69]. The up-regulation of miR-34a inhibits Notch signalling and promoted CSCs differentiation. On the contrary, a low miR-34a level could induce the activation of Notch signalling and increase the proliferation of colorectal CSCs [69].

Most importantly, the interactions of miRNAs with signalling pathways can regulate the therapy resistance property of CSCs derived from many malignancies, including colon CSCs [70,71]. For example, miR-140 was up-regulated, whereas miR-215 was down-regulated in CD44+/CD133+ colon CSCs [72]. miR-140 confers a methotrexate (MTX) and 5-FU resistant phenotype through targeting histone deacetylase 4 (HDAC4) in colorectal CSCs [72], while miR-215 increased the sensitivity of this cell population to chemotherapeutic drugs (methotrexate and tomudex), by targeting denticleless protein homolog (DTL) [73]. Furthermore, polycomb complex protein (BMI1) was identified as a target of miR-215. It is involved in a feedback loop of miR-215, the cell cycle and stemness gene *caudal-type homeobox 1* (*CDX1*). In this loop, miR-215 could inhibit the expression of *CDX1*, whereas CDX1 directly activated miR-215 transcription in CRC cells. It is a key transcription factor in regulating differentiation of normal colon cells, as well as cancer cells. miR-215 transcript was not found in colon CSCs when compared to the non-CSCs colon carcinoma cells. The ectopic expression of miR-215 resulted in decreased clonogenicity in poorly differentiated adenocarcinoma cells. On the other hand, miR-215 knockdown enhanced clonogenicity and impaired differentiation in CDX1 expressing cells [74]. Thus, CDX1/miR-215/BMI1 axis was demonstrated in governing the development and survival of colon CSCs.

Several other miRNAs such as miR-203, miR-328 and miR-497, have also been identified in the regulation of stemness and chemoresistance in colon carcinoma [75,76,77]. The down-regulation of miR-203 in CD44+ cells derived from colon carcinoma (HCT-15 and HCT-116) could inhibit the stemness of CRC cells by targeting Snail signalling [77]. Snail is a family of transcription factors that promote the repression of the adhesion molecule, E-cadherin, to regulate epithelial to mesenchymal transition (EMT) during embryonic development.miR-328 was down-regulated in stem-like side population (SP) cells in CRC, while an overexpression of miR-328 resulted in the inhibition of SP cells [76]. In addition, the exogenous overexpression of miR-328 sensitized CRC cells to chemotherapeutic agents by directly suppressing ATP-binding cassette super-family G member 2(ABCG2) and matrix metallopeptidase 16 (MMP16) [76]. Similarly, miR-497, another example of miRNA, is down-regulated in CRC and involved in cell proliferation, invasion, and the sensitivity to cisplatin and 5-FU [77].

Therefore, targeting miRNAs expression or exogenous supplementation of their analogues might be a useful strategy for eradicating CSCs, re-sensitizing drug-resistant cells to anti-cancer agents, and reducing the chance of cancer recurrence.

## 6. Quiescence of CSCs and Therapy Resistance

A growing body of information suggests that CSCs’ quiescence may contribute to therapy resistance, as some of the cytotoxic agents target only highly proliferating cancer cells [78,79,80]. These quiescent CSCs can re-enter the cell cycle after treatment and can accelerate tumour regeneration by activating cell growth and proliferative signalling pathways [11,81]. Indeed, the treatment of colon CSCs with 5-FU resulted in a reversible quiescent G_0_ state with overexpressed tyrosine kinase c-Yes [41]. This c-Yes expression is associated with the dormancy of colorectal CSCs [3]. c-Yes maintains the balance between quiescent and 5-FU resistant carcinoma cells by regulating the cytoplasmic/nuclear ratio of the Yes-associated protein (YAP) transcription co-activator [41]. Furthermore, a high-mobility group A1 (HMGA1) protein was down-regulated in colon CSCs in the quiescent state [82]. HMGA1is a master regulator in both CSCs [83,84] and normal embryonic stem cells [84,85]. HMGA1 is overexpressed in colon CSCs when compared to non-neoplastic colon mucosa and colon carcinoma tissues [82]. However, the silencing of HMGA1 enhanced stem cell quiescence and decreased self-renewal and sphere-forming efficiency of CSCs. Furthermore, HMGA1 transcriptionally regulates p53, which is known to control the balance between symmetric and asymmetric divisions in CSCs [82]. Thus, the relationship between p53 and HMGA1 suggested that HMGA1 plays a significant role in regulating both self-renewal and the symmetric/asymmetric division ratio in CSCs. Therefore, blocking HMGA1 function could be an effective anti-cancer therapy.

Zinc Finger E-Box Binding Homeobox 2(ZEB2) is a transcription factor up-regulated in colorectal CSCs [86]. ZEB2 is involved in stem cell plasticity and epithelial-mesenchymal transition (EMT) and maintains the expression of anti-apoptotic factors, such as pCRAF and pASK1 [86]. ZEB2 overexpression resulted in up-regulation of pCRAF/pASK1, which subsequently caused increased chemoresistance, enhanced stemness/EMT traits and slowed down the proliferation of tumour xenografts [86]. Thus, the activation of the ZEB2/pCRAF/pASK1 axis induced the increase prevalence of quiescent cancer stem cells (QCSCs), with a stemness/EMT phenotype followed by the chemotherapy treatment of tumour xenografts, especially in a chemotherapy-quiescent state. Furthermore, increased ZEB2 level was found to be associated with lower relapse-free survival of patients with CRC [86]. Therefore, designing and developing strategies to identify molecular signatures associated with these quiescent and therapy-resistant CSC populations could improve the survival of patients with CRC.

## 7. Metabolic Adaptation of CSCs Facilitates Therapy Resistance

Accumulating evidence suggests that chemotherapeutic insults can induce changes in colon cancer cells’ metabolism, which subsequently results in the reduction of drug efficacy. The majority of cancer cells, in order to give an advantage in their growth, divert metabolites into anabolic pathways through Warburg effects [87]. Treatment on colon cancer cells with 5-FU induces a metabolic switch in mesenchymal stem-like cells to oxidative phosphorylation (OXPHOS) phenotype by metabolic reprogramming, to meet energy demands [87]. Moreover, 5-FU resistant carcinoma cells showed a de novo expression of pyruvate kinase M1 (PKM1) and the repression of pyruvate kinase M2 (PKM2), correlating with the repression of the pentose phosphate pathway by decreasing the nicotinamide adenine dinucleotide phosphate (NADPH) level. However, in antioxidant defences, where 5-FUresistant cells increase production of ROS, promote PKM2 oxidation and induce stemlike phenotype by expressing stemness associated markers, increasing self-renewal capacity, and the formation of anchorage-independent spheres [87]. Similarly, the response to 5-FU in a xenotransplantation model of human colon carcinoma confirms the activation of mitochondrial function [87]. Evidently, the combination of 5-FUwith a pharmacological inhibitor of OXPHOS inhibited the spherogenic potential of colon carcinoma cells and diminished the expression of stem-like markers. These findings demonstrated the necessity of targeting the adapted phenotype of CSCs to achieve highest therapeutic outcome in clinical settings.

Additionally, the chemo-treatment of patient-derived colonosphere cultures resulted in an increased mitochondrial biomass, increased expression of respiratory chain enzymes and higher rates of oxygen consumption, indicating a chemotherapy-induced metabolic shift towards OXPHOS from glycolysis [88]. Further study suggested that a histone deacetylase namely sirtuin-1 (SIRT1) and its transcriptional co-activator peroxisome proliferator-activated receptor gamma coactivator 1-α (PGC1α) induce the oxidative phosphorylation of colonospheres [88,89,90]. The activation of SIRT1 causes the induction of oxidative phosphorylation, which enables cancer cells to become resistant to non-lethal chemotherapy drugs (a hallmark of CSCs). Chemotherapy on CRC induces a SIRT1/PGC1αdependent increase in OXPHOS that increases the survival of carcinoma cells [88]. The knockdown of SIRT1 or PGC1α inhibited chemotherapy-induced OXPHOS and significantly sensitized patient-derived colonospheres, as well as tumour xenografts to chemotherapy. A similar phenomenon was noted in chemotherapy-exposed resected liver metastases [88], which further indicated that chemotherapy-induced changes in tumour metabolism potentially affect drug efficacy in patients with cancers.

## 8. Therapeutic Modelling of Colorectal CSCs to Inhibit Therapy Resistance

**Figure 4:**
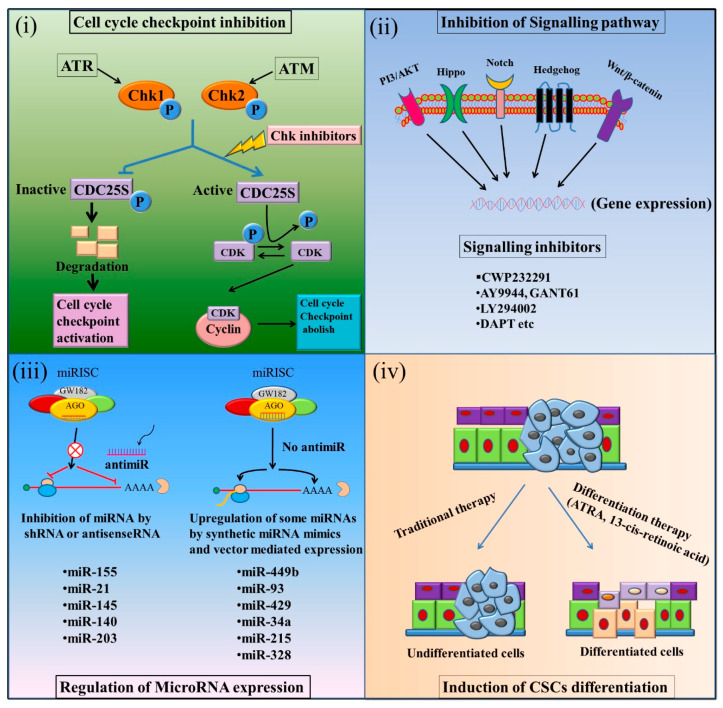
Therapeutic modelling to reduce or inhibit the therapeutic resistance property of CSCs. (**i**) Inhibition of cell cycle checkpoints, (**ii**) Inhibition of hyper-activated signalling pathways, (**iii**) Regulated expression of miRNAs, (**iv**) Induction of differentiation of CSCs.

Targeting CSCs has opened a new horizon to overcome chemo–radiotherapy resistance of cancers [91,92,93]. Many strategies, including the induction of the differentiation of CSC, the suppression of specific signalling or metabolic pathways, inhibiting cell cycle progression by inhibitors, using miRNA expression to aid conventional therapies etc., have already been proposed (Figure 4).

Non-CSC cancer cells have no self-renewal ability, cannot proliferate indefinitely, and most importantly, are more susceptible to conventional therapies than CSCs. Therefore, the differentiation of CSCs into mature cancer cells might be an attractive option to treat patients with CRC. For example, patients with promyelocytic leukaemia treated with retinoic acid, an inducer of CSC differentiation, have showed promising clinical outcome [92]. Treatment with retinoic acid increased the intracellular concentration of retinoic acid, which in turn causes up-regulation of its retinoic acid receptor (RAR), resulting in the reduced activity of a cancer-mutated receptor, promyelocytic leukaemia- retinoic acid receptor (PML-RAR) [92]. Therefore, retinoic acid acts as an agonist of steroid hormone receptors and induces the differentiation of abnormal cancer cells, thereby increased the therapeutic sensitivity.

Several strategies had shown potential in eradicating CSCs by targeting the activated growth and survival signalling [17,94,95,96]. For example, an antiparasitic drug, pyrviniumpamoate, was shown to inhibit LRP6-mediated axin degradation and inhibited the potency of β-catenin stabilization [94]. Moreover, the pyrvinium treatment of colon carcinoma cells (HCT116 and SW480) with mutated *adenomatous polyposis coli (APC)* or β-catenin (*CTNNB1)* suppressed cell proliferation via inhibiting Wnt signalling [94]. Additionally, the allosteric activation of casein kinase1α (CK1α) could cause the inhibition of Wnt signalling [95]. Furthermore, the Wnt pathway can be regulated by Notch signalling, since a group of Wnt/β-catenin downstream genes is directly regulated by Notch [95]. During inactivation of β-catenin signalling, these genes were up-regulated by active Notch1expression.On the other hand, γ-secretase inhibitors inhibited these genes, resulting in reduced cells proliferation and survival [95]. Thus, the expression of activated Notch1 resulted in the partial reversion of blocking Wnt/β-catenin pathway. A subpopulation of CD133+, CD44+ CSCs cells derived from colon cancer cells (HCT116), resistant to 5-FU and oxaliplatin, are sensitive to γ-secretase inhibitor (DAPT). Treatment of these CSCs phenotypic cells with DAPT decreased in vitrocells’ growth and suppressed growth of tumours in animal model [17]. Moreover, γ-secretase inhibitors’ mediated inactivation of Notch1 signalling could increase the sensitivity of cancer cells to conventional chemotherapeutics [96]. Metformin, a promising compound, combined with conventional chemotherapeutics, has recently been identified as a potential and attractive anticancer adjuvant drug. Metformin improves the efficacy of conventional therapies and decreases chemotherapeutic doses. It mediates its action through insulin-dependent and AMP-activated protein kinase (AMPK)-dependent effects, by selectively targeting CSCs, reversing multidrug resistance and inhibiting tumour metastasis and acts on *mammalian target of rapamycin* (mTOR) pathway inhibition [97,98,99]. Therefore, inhibiting signalling pathways, such as Wnt pathway and Notch pathway, may be effective strategies for targeting colon CSCs and overcoming the resistance to conventional chemotherapeutics.

With the increasing understanding of colorectal CSCs mediated therapy resistance by several other mechanisms, little is known about how the metabolic switching of CSCs fuels therapy resistance. There are only a few reports concerning metabolic adaptations of colon CSCs and overcoming resistance to chemotherapy [89,100,101,102,103]. The overexpression of silent mating type information regulation 2 homolog 1 (SIRT1) in 5-FU resistant carcinoma cells has been implicated for the promotion of carcinogenesis and the development of drug resistance [100]. SIRT1 is a NAD+-dependent histone deacetylase, which contributes in metabolic switching to OXPHOS. Moreover, SIRT1 has been found to regulate various cellular processes, including cell survival under genotoxic and oxidative stress [101,102]. A recent meta-analysis suggested that SIRT1 expression could be regarded as a negative prognostic marker of patients with CRC [102]. SIRT1 maintains stem-like features of colon CSCs and is co-expressed with the CD133 [89]. The deficiency of SIRT1suppressed the number of CD133+ cells and reduced tumourigenicity, colonies, and spheres formation capacity of colon CSCs [89]. Interestingly, miR-34 regulates the expression of SIRT1, and therefore, could be utilized against SIRT1 [100,103]. The treatment of 5-FU resistant colon carcinoma cells (DLD-1) with miR-34a directly inhibit SIRT1 expression which results in the suppression of 5-FU with the help of reduced E2F family proteins expression [100,103]. In addition, the knockdown of *SIRT1* gene in colon adenocarcinoma cells (SW620) suppressed the expression of several stemness-associated genes, such as Oct4, Nanog, and Tert [89]. Therefore, in order to sensitize the cancer to 5-FU therapy, targeting the *SIRT1* gene could be a promising option, as reduced expression of SIRT1 further results in p53-mediated apoptosis [103]. These findings suggest that SIRT1 can be considered as a novel predictive marker or a new target for therapy for CRC.

The inhibition of cell cycle checkpoint proteins could be a potential approach to overcome the resistance of CSC to conventional cancer chemotherapy. Cells with aberrantly activated checkpoints lead to proliferation in an uncontrolled manner, causing genomic and metabolic destabilization and subsequently leading to increase cell mass, i.e., cancer generation. Several abnormal activations in intracellular signalling molecules and/or transcription factors are associated with cell cycle checkpoints dysregulation, which subsequently results in cancer development. Flavonoid morin and telomerase inhibitor (MST-312) are two potential therapeutic compounds which have been found to lower tumourigenicity of CSCs by targeting signal transducer and activator of transcription 3 (STAT3) and telomerase in human colon cancer cells [104]. A combination of morin and MST-312 inhibited the phosphorylation of cellular proteins, such as p53 and checkpoint kinase 2 (Chk2), the two important cell cycle proteins, which play vital roles in DNA damage checkpoint control [104]. A dose dependent inhibition of proliferation of CD44+ CSCs was noted after morin and MST-312 treatment [104]. Colon cancer cells harbouring a mutated *APC* gene are resistant to treatment of 5-FU. Deactivation of checkpoint kinase 1 using antisense siRNA-mediated knockdown sensitized these *APC*-mutated colon cancer cells to the treatment of 5-FU [105]. Therefore, cancer cells with enriched CSCs phenotype could be more susceptible to apoptosis when they lack the activity of cell cycle regulating proteins, such as checkpoint kinase 1. p21-activatedkinase 1 (PAK1), another cell cycle regulator, may act as an activator of Wnt/β-catenin pathway, and therefore can be a potential target for treatment of CRC. A synergistic effect of PAK1 inhibitor PF-3758309 and 5-FU has already been observed in the cellular and animal model of CRC [106]. In vitro and in vivoxenograft tumour models suggested thatPAK1 plays crucial roles in maintaining stem-cell-like features, such as the expression of CD44, tumourigenicity, and spherogenicity of CSCs in CRC [106].

MiRNAs have the potential to be used as novel targets to re-sensitize drug-resistant colon CSCs to anticancer therapies. Studies are being conducted, using several miRNAs as proof-of-concept. For example, exogenous manipulations of miR-7, miR-22, miR-34a, miR-122, miR-140, miR-143, miR-215, miR-224 and miR-320 in CRC have demonstrated significant therapeutic and/or chemo-sensitizing effects [73,107,108,109,110,111,112,113,114]. Lactate dehydrogenase A (LDHA), a target of miR-34a, is significantly elevated in 5-FU resistant colon carcinoma cells. The ectopic overexpression of miR-34a resulted in re-sensitization of 5-FU-resistant carcinoma cells to 5-FU, followed by LDHA suppression [109]. MiR-22, through activating phosphatase and tensin homolog (PTEN) signalling, could re-sensitize paclitaxel-resistant p53 mutated colon cancer cells to paclitaxel [115]. Similarly, miR-122 and miR-320 have re-sensitizing 5-FU-resistant colon carcinoma cells to 5-FU, by modulating pyruvate kinase isozymes 2(PKM2) and forkhead box M1 (FOXM1) actions respectively [111,113]. Moreover, targeting oncogenic miRNAs by antisense miRNAs might be another promising way to reverse drug resistance (Figure 4). For example, miR-23a and miR-21, two of the most aberrantly expressed miRNAs in 5-FU resistant colon cancer cells, could be subjected to such therapeutic strategies. MiR-23a inhibited the expression of apoptotic protease activating factor1 (APAF-1), along with two important caspases (caspases-3 and -7) [112]. The exogenous supplementation of miR-23a antisense into 5-FU resistant carcinoma cells increased APAF-1 level and induced the enhanced activation of caspase-3 and -7, thereby increased 5-FU mediated apoptosis in these cells [112].

The therapeutic strategies aiming for molecular targeting of colorectal CSCs are going on. For example, a clinical trial (NCT01440127) targeting colorectal CSCs with metformin, a drug used for the treatment of patients with diabetes is used to determine the difference between treating patients with and without metformin targeting cancer stem cells. However, the outcome of the study is yet to come. Another phase I/II clinical trial (NCT02176746) of active immunotherapy for colorectal CSCs using a CSC-loaded dendritic cell as vaccine has been reported. CSCs are more effective in inducing anti-tumour immunity than unselected tumour cells, as CSCs act as more immunogenic antigens. CSC-vaccinated hosts contain high levels of IgG bound to CSCs, which subsequently results in the lysis of CSC by complement activation. Moreover, cytolytic T lymphocytes (CTLs) from peripheral blood mononuclear cells or splenocytes harvested from CSC-vaccinated hosts could kill CSCs in vitro. Therefore, CSC-primed antibodies and T cells selectively target CSCs and confer anti-tumour immunity. Together, these results provide an option to develop a new type of cancer immunotherapy, based on the formation of CSC vaccines that can specifically target CSCs.

## 9. Concluding Remarks

Accumulating information in cancer research indicates that CSC is the root of cancer progression and therapy resistance. The current conventional chemo-radiotherapies target the differentiated cancer cells; thereby, CSCs remain unharmed by their therapy resistance property. Thus, in-depth knowledge of the biology, function, and clinical implications of CSCs in colorectal cancer therapy resistance is imperative to develop effective therapeutic modalities for the patients with colorectal cancer. In this review, we have summarized how colorectal CSCs gain therapy resistance and how this malicious property enables CSCs to escape therapeutic insults. We also outline some potential options to overcome therapy resistance. It is suggested that creating combined therapy regimens, in which conventional drugs are supplemented with novel CSC-targeting drugs, might offer improved overall and cancer-free survival rates for patients with CRC. A potential dose reduction of conventional chemotherapeutics could help limit their toxicity and improve patients’ quality of life.

## Figures and Tables

**Table 1:** Genes/Transcription factors/Proteins associated with signalling pathway mediated drug resistance in cancer stem cells in colon carcinoma.

Genes/Transcription Factors/Proteins	Functions	Signalling Pathways	Reference
ABCG2	A Wnt/β-catenin pathway mediated regulation of ABCG2 by miR-199a/b contributes to cisplatin resistance in colon carcinoma	Wnt/β-catenin	[14]
LGR5	Promotes Wnt signalling through the neutralisation of two transmembrane E3 ligases, RNF43 and ZNRF3. These enzymes remove Wnt receptors from the cell surface, thus negatively regulate Wnt signalling	Wnt/β-catenin	[29]
CD44, CD24, CD133, and EpCAM	Expression of these markers are important in Wnt mediated CSCs growth and therapy resistance in colon carcinoma	Wnt/β-catenin	[30,31,32,33]
APC	Restoration of mutated APC in colon carcinoma results in rapid and widespread tumour-cell differentiation and maintained inhibition without relapse of carcinoma	Wnt/β-catenin	[34]
β-catenin	Regulates the transcription of downstream target genes to increase stem cell characteristics and subsequently therapy resistance in colon carcinoma	Wnt/β-catenin	[34]
Wnts	Aberrant expression of Wnt proteins results in hyper activation of Wnt/β-catenin and consequently propagates stem cell characteristics	Wnt/β-catenin	[34]
Notch1	Higher expression of Notch 1 increases CSCs phenotype and as a result positively regulates the number of colon cancer cells resistant to therapy	Notch	[17]
Hes 1	Induction of Hes 1 protects cancer stem cells from differentiation	Notch	[17]
GLI-1	Promotes Hh mediated stem cell characteristics of colon carcinoma cells and subsequently increases therapy resistance of colon carcinoma	Hedgehog	[39]
Yes-associated protein 1 (YAP1)	High expression of YAP target genes in the tumour was coupled increased therapy resistance of colon carcinoma and poor survival of patients	Hippo	[40]
YES1	YES1 regulates drug resistance of colon carcinoma through regulation of YAP1 expression	Hippo	[41]
MACC1	Promotes sphere formation, and increases the expression levels of pluripotent markers: CD44, CD133 and Nanog	PI3K/AKT	[45]

**Table 2:** miRNAs associated with the therapy resistance property of colorectal CSCs.

MicroRNAs	Expression Pattern in Cancer Cells	Targets	Functions	Reference
miR-449b	Down-regulated	Cyclin D1 and E2F3	Inhibits colon CSCs proliferation	[60]
miR-93	Down-regulated	HDAC8 and Transducin like enhancer of Split-4 (TLE4)	Inhibits colon CSCs proliferation by suppressing Wnt/β-catenin signalling pathway	[61]
miR-155	Up-regulated	RhoA	Plays an important role in TGF-beta-induced EMT and cell migration and invasion by targeting RhoA	[64]
miR-429	Down-regulated	ZEB1 and ZEB2	Causes up-regulation of E-cadherin and inhibits EMT process	[65]
miR-451	Down-regulated	Macrophage migration inhibitory factor (MIF)	Sensitizes colospheres to irinotecan and reduces the capacity of self-renewal and tumorigenicity	[66]
miR-21	Up-regulated	TGF-βR2	miR-21 might cause activation of Wnt signalling pathway with an increase in β-catenin levels, TCF/LEF activity, and the expression of Wnt targeted genes *c-Myc* and *CCND1*	[67]
miR-145	Up-regulated	Unknown	Correlates with miR-21 to maintain CSCs proliferation and/or differentiation, therefore contributes in the development of chemoresistance	[68]
miR-34a	Down-regulated	Notch1	Regulates the proliferation and differentiation of colorectal CSCs by targeting Notch signalling	[69]
miR-140	Up-regulated	HDAC4	Confer an MTX and 5-FU resistant phenotype through targeting	[72]
miR-215	Down-regulated	Denticleless protein homolog (DTL)	Increases the sensitivity to methotrexateand tomudex	[73]
miR-203	Up-regulated	Snail	Increases stemness and chemoresistance by targeting Snail signalling	[75]
miR-328	Down-regulated	ABCG2 and matrix metallopeptidase 16 (MMP16)	Overexpression of miR-328 results in inhibition of SP cells and most importantly sensitizes colorectal cancer cells to chemotherapeutic agents	[76]
miR-497	Down-regulated	Insulin-like growth factor 1	Regulates proliferation, invasion, and the sensitivity to cisplatin and 5-FU	[77]

## Abbreviations

CRC	Colorectal cancer
CSCs	Cancer stem cells
5-FU	5-Fluorouracil
MiRNA	MicroRNAs
CD133	Cluster of differentiation 133
CD44	Cluster of differentiation 44
CD24	Cluster of differentiation 24
CD166	Cluster of differentiation 44
ESA	Epithelial surface antigen
Lgr5	Leucine-rich repeat-containing G-protein coupled receptor 5
APC	Adenomatous polyposis coli
TCF	T-cell factor
YAP1	Yes-associated protein 1
EMT	Epithelial mesenchymal transition
RTKs	Receptor Tyrosine Kinases
TEAD1	TEA domain family member 1
CYR61	Cysteine-rich angiogenic inducer 61
ANKRD1	Ankyrin Repeat Domain 1
*MACC1*	*Metastasis-associated colon cancer 1*
DSBs	Double-strand breaks
DDR	DNA damage response
ERCC1	Excision repair cross-complementing group 1
ROS	Reactive oxygen species
*CCND1*	*Cyclin D1*
TLE4	Transducinlike enhancer of Split
PDCD4	Programmed Cell Death Protein 4
MTX	Methotrexate
FACS	Fluorescence-activated cell sorting
MMP6	Matrix metallopeptidase 16
HMGA1	high-mobility group A1
SFE	Sphere-forming efficiency
PKM1	Pyruvate kinase M1
PKM2	Pyruvate kinase M2
SIRT1	Sirtuin-1
PGC1α	Peroxisome proliferator-activated receptor γ coactivator 1α
RA	Retinoic acid
RAR	Retinoic acid receptor
STAT3	Signal transducer and activator of transcription 3
Chk2	Check point kinase 2
FOXM1	Forkhead box M1
AMPK	AMP-activated protein kinase
